# Integrated Anode Electrode Composited Cu–Sn Alloy and Separator for Microscale Lithium Ion Batteries

**DOI:** 10.3390/ma12040603

**Published:** 2019-02-18

**Authors:** Yuxia Liu, Kai Jiang, Shuting Yang

**Affiliations:** 1 School of Chemistry and Chemical Engineering, Henan Normal University, Xinxiang 453007, China; lyxia159951@163.com; 2 Henan Chemical Industry Research Institute Co. Ltd., Zhengzhou 450052, China

**Keywords:** electrodeposition, separator, Cu–Sn alloy, anode, integration

## Abstract

A novel integrated electrode structure was designed and synthesized by direct electrodepositing of Cu–Sn alloy anode materials on the Celgard 2400 separator (Cel-CS electrode). The integrated structure of the Cel-CS electrode not only greatly simplifies the battery fabrication process and increases the energy density of the whole electrode, but also buffers the mechanical stress caused by volume expansion of Cu–Sn alloy active material; thus, effectively preventing active material falling off from the substrate and improving the cycle stability of the electrode. The Cel-CS electrode exhibits excellent cycle performance and superior rate performance. A capacity of 728 mA·h·g^−1^ can be achieved after 250 cycles at the current density of 100 mA·g^−1^. Even cycled at a current density of 5 A·g^−1^ for 650 cycles, the Cel-CS electrode maintained a specific capacity of 938 mA·h·g^−1^, which illustrates the potential application prospects of the Cel-CS electrode in microelectronic devices and systems.

## 1. Introduction

With the development of the electronics industry and micro-machining technology, microelectronic devices have been well-researched with applications in many areas, such as pacemakers, miniature transmitters, sensors, hearing aids, defibrillators, etc. The trends for these devices are towards miniaturization and integration on a millimeter scale [1,2]. Clearly, traditional power supplies with their cumbersome and bulky size cannot meet the increasing demands of microelectronic devices and systems. Therefore, it is essential to develop micro-power supplies. Higher power and higher energy on a smaller scale are the main goals of micro-electromechanical systems (MEMS) technology [3]. Chen et al. [4] recently designed micro-batteries (MB306) with high specific capacity for a juvenile salmon acoustic telemetry system (JSATS). Panasonic [5] commercialized the smallest pin-type batteries in wearable devices, electronic pens and medical devices. Micro-scaled all-solid-state thin-film lithium ion batteries (MLIBs) have attracted many people’s attention because of their high energy density, high open circuit voltage, long cycling life, good safety performance and ease of integration [6,7,8,9]. To realize energy miniaturization and integration, the best options for MLIBs are based on the intrinsic electrical properties of the materials and electrode structure.

Owing to the high theoretical capacity of 992 mA·h·g^−1^ (Li_22_Sn_5_), low platform potential (0.3–0.6 V) and low cost, Sn-based anode electrodes have been researched widely as one of the most promising anode electrodes for microscale lithium ion batteries (LIBs) [10,11,12,13,14,15]. However, the fast capacity decay caused by the severe volume expansion effect (about 300%) and the pulverization-induced loss of active material has limited the practical application of Sn-based anodes. Nanocrystalline and composite can be an effective way to overcome these drawbacks and enhance the cycle stability [16,17,18,19]. A water-soluble conductive binder studied by Xing et al. [20] improved the specific capacity of nanosized Sn anodes to 593 mA·h·g^−1^ after 600 cycles at 500 mA·g^−1^. Zhang et al. [21] prepared a 3D nanoporous Cu_6_Sn_5_/Cu composite by one-step dealloying of Al_10_Cu_3_Sn, which showed a capacity of 326 mA·h·g^−1^ after cycling at 100 mA·g^−1^ for 50 cycles. A Sn–Fe@C network synthesized by Shi et al. [22] exhibited a reversible capacity of 441.6 mA·h·g^−1^ after 100 cycles at 100 mA·g^−1^. Though all these methods improve the cycle performance of Sn-based anodes to an extent, integration compatibility is still the main issue to consider.

At present, the integrated electrode is the main method used to improve integration compatibility, which saves time and cost in the mixing and coating production process. Lou et al. [23] prepared hierarchical Co_x_Mn_3−x_O_4_ array micro-/nanostructures grown on stainless steel by a solvothermal route and a subsequent annealing treatment as promising integrated binder-free electrodes for MLIBs. Guo et al. [24] fabricated Cu_2_ZnSnS_4_ thin film with radio frequency magnetron sputtering and applied it in microscale thin-film LIBs. In our previous work [25], multi-scale Cu–Sn alloy film with superior cycle stability was prepared as the anode electrode directly by electro-deposition. All the integrated electrodes exhibit superior cycle performance; however, the complex assembly process is still a big obstacle to practical application. To further simplify the manufacturing process and improve the integration compatibility of MLIBs, a novel integrated structure electrode, electrodepositing Cu–Sn alloy anode materials on the solid electrolyte membrane, was proposed and designed. Since it is difficult to find a commercial solid-state electrolyte membrane, this idea had to be tested on the separator.

In this work, a novel integrated structure electrode was designed and synthesized by directly electrodepositing Cu–Sn alloy anode materials on the Celgard 2400 separator (Cel-CS electrode). Firstly, directly electrodepositing Cu–Sn alloy anode materials on the separator can greatly simplify the fabrication process and improve the integration compatibility of MLIBs. Secondly, by abandoning the traditional Cu current collector, the Cel-CS electrode not only increases the energy density of the whole electrode, but also buffers the mechanical stress caused by the volume expansion of Cu–Sn alloy active material, thus effectively preventing active material from falling off from the substrate and improving the cycle stability. The Cel-CS electrode exhibits excellent cycle performance and superior rate performance. A capacity of 728 mA·h·g^−1^ can be achieved after 250 cycles at the current density of 100 mA·g^−1^. Even cycled at the current density of 5 A·g^−1^ for 650 cycles, the Cel-CS electrode could still show a specific capacity of 938 mA·h·g^−1^, which illustrated the potential application prospects of Cel-CS electrode in microelectronic devices and systems.

## 2. Experiment

### 2.1. Preparation of the Integrated Electrode

The separator used in this work is commercial Celgard 2400 (Celgard, Concord, NC, USA). A conductive nano-carbon layer (about 5 nm) needs to be sprayed on the separator in advance by magnetron sputtering. The Cu–Sn alloy layer growth on the pretreated separator under appropriate electro-deposition conditions. The composite electrodes were prepared with DC current deposition by constant voltage electro-deposition. The electro-deposition process and parameters have been discussed in our previous work [25]. Electro-deposition reaction occurred in an electrolytic cell, and the titanium plate and separator after pretreatment were regarded as anode and cathode, respectively. The distance between the two polar plates was 4 cm, and the volume of the electroplating solution was 500 mL. The electroplating solution was made by mixing 100 g K_4_P_2_O_7_·3H_2_O, 6.2 g SnCl_2_·2H_2_O, 4.7 g CuCl_2_·2H_2_O and 5 mL electroplating refiner (a mixture of glucose, gelatin, glutamic acid, and PEG) in deionized water. The integrated electrode was obtained by keeping the voltage constant at 7 V for 300 s. The novel integrated electrode which composited anode material with the Celgard 2400 separator (Cel-CS electrode) was obtained. As a contract, the Cu–Sn alloy layer composite with the Cu collector (Cu-CS electrode) was also prepared in the same electroplating conditions. The loading of the Cu–Sn alloy layer was approximately 1.7 mg·cm^−2^.

### 2.2. Characterization

The phase structure of the Sn–Cu alloy anode was determined by X-ray diffraction (XRD) on a D8 Advance diffractometer (Bruker, Karlsruhe, Germany). The morphologies and microstructures of the Sn–Cu alloy anode before and after cycling were characterized by using a field emission scanning electron microscope (FESEM, JSM-6700F, JEOL, Tokyo, Japan) equipped with an energy dispersive spectros-copy (EDS). The XPS was performed by a Perkin-Elmer PHI 5000C ESCA system (Perkin-Elmer, Waltham, MA, USA) with Al Kα radiation operated at 250 W.

### 2.3. Electrochemical Measurements

Electrochemical performances of the Cel-CS electrode were measured using CR2032 coin-type half cells. The prepared Sn–Cu alloy electrode was cut into discs (12 mm in diameter) as work electrodes. Li foil was used as the counter and reference electrode, and 1.0 M LiPF_6_ in ethylene carbonate/dimethyl carbonate (EC/DMC = 1:1 by volume) as the electrolyte. Galvanostatic charge/discharge profiles and rate capability between 0.01 and 3.0 V (vs. Li^+^/Li) at various current densities were measured on a LAND Cell test system (CT2001A, Wuhan, China). Cyclic voltammograms (CV) and electrochemical impedance spectroscopy (EIS) were carried out on the CHI760E (Shanghai, China). CV tests were performed between 0.01 and 2.5 V (vs. Li^+^/Li) at a scan rate of 0.2 mV·s^−1^. The EIS data were tested between frequencies of 0.01 Hz and 100 kHz at a signal amplitude of 5 mV.

## 3. Results and Discussion

**Figure 1:**
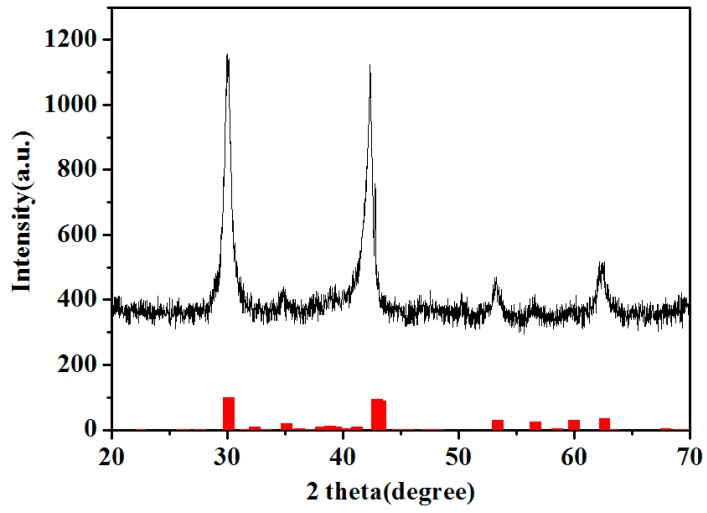
The XRD patterns of the Cel-CS electrode.

Figure 1 illustrates the XRD patterns of the Cel-CS electrode. All the diffraction peaks can be assigned to the Cu_6.5_Sn_5_ (Hexagonal, P63/mmc (194), JCPDS card NO. 47-1575) phase, which was the main component of the electroplating Sn–Cu alloy.

**Figure 2:**
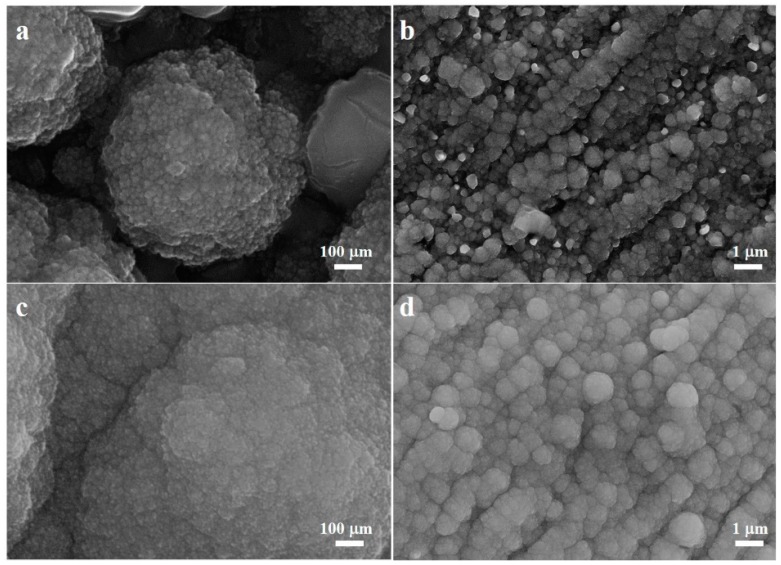
The FESEM pattern of the Cel-CS electrode (**a**,**b**) and the Cu-CS electrode (**c**,**d**) with different magnifications.

Figure 2a–d show the morphology of the Cel-CS electrode and the Cu-CS electrode with different magnifications. It can be seen that the deposited Cel-CS layer was composed of the multi-scaled particles. Nanoparticles aggregated together to form secondary-grade micro-sized spherical particles, and secondary-grade spherical particles of Cu–Sn alloy stacked with each other to form the layered structure. The morphology of the Cu–Sn alloy layer with the traditional Cu collector (Figure 2c,d) was similar to the Cel-CS electrode, except the grain size of the secondary particle was larger.

**Figure 3:**
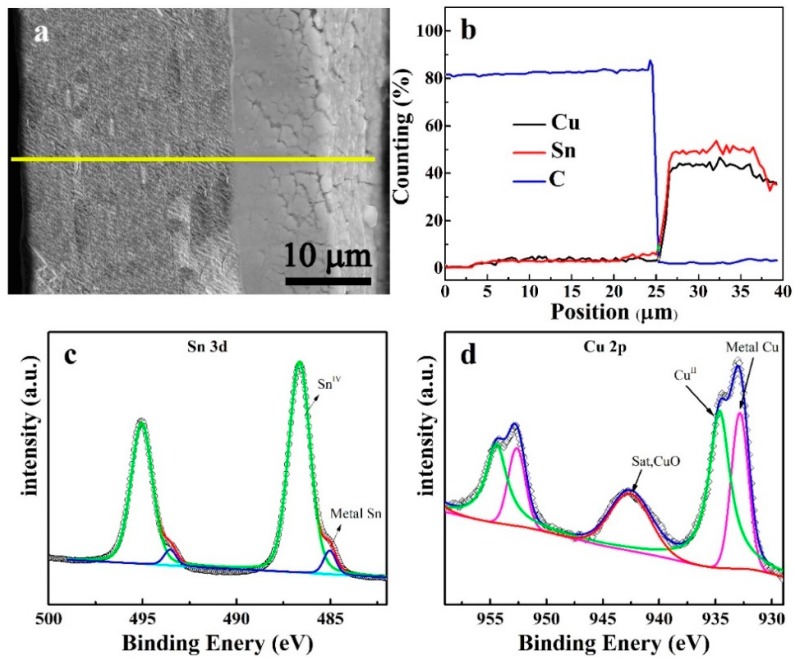
Cross-section image (**a**) and the linear scan of the EDS result (**b**) of the Cel-CS electrode. The high-resolution XPS survey of Sn 3d (**c**) and Cu 2p (**d**).

The linear scan of the EDS result of Cel-CS electrode is shown in Figure 3a. The thickness of the separator was about 25 μm, and of the electrodeposited layer was about 10 nm. The content ratio of Cu and Sn (Figure 3b) was about 1.1, which is close to the component of Cu_6.5_Sn_5_. The surface element state of the Cel-CS electrode was measured by XPS. The high-resolution Sn 3d profiles (Figure 3c) were fitted to 485 eV and 486.6 eV, which can be assigned to the typical oxidation state of Sn^0^ and Sn^4+^, respectively. Figure 3d is the high-resolution Cu 2p spectrum. The fitted peaks at 932.5 eV corresponded to Cu^0^, and the peak located at the binding energy of 935.6 eV was characteristic Cu^2+^, while 941.9 eV was attributed to the shake-up satellite structure. Consistent with the results of EDS, the atomic ratio of Cu and Sn which was calculated by the peaks’ area of XPS was also about 1.1.

**Figure 4:**
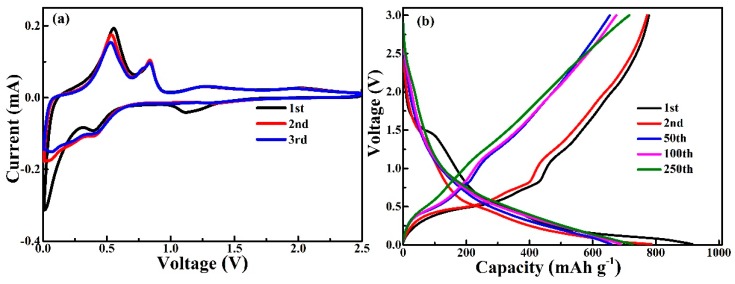
The cyclic voltammograms (**a**) and discharge-charge curves at different cycles (**b**) of the Cel-CS electrode.

Cyclic voltammograms (CVs) of the Cel-CS electrodes between 0.01 and 2.5 V (vs Li^+^/Li) at 0.2 mV·s^−1^ are illustrated in Figure 4a. In the first discharge process, three reduction peaks were observed at 1.0 V, 0.6 V, and 0.1 V, corresponding to the formation of a solid electrolyte interface (SEI) film layer, the transformation of η’-Cu_6_Sn_5_ into Li_2_CuSn-type phase, and the alloying process involved the lithiation of Sn to finally yield Li_4.4_Sn from Li_2_CuSn, respectively [26,27,28,29]. In the first anodic scan, the peak located at 0.6 and 0.8 V can be assigned to the reversible de-alloying reaction of Li_4.4_Sn to Li_2_CuSn and Cu_6_Sn_5_. In the subsequent cycles, the two cathodic peaks change to board slope at 0.5 and 0.1 V, which can be attributed to the structure transformation and recombination [16,26,30,31]. The curves for the second and third cycles were nearly overlapping, and implied good reversibility from the 2nd cycle. Figure 4b shows the 1st, 2nd, 50th, 100th, and 250th galvanostatic discharge/charge (GDC) curves of the Cel-CS electrode at the current densities of 200 mA·g^−1^ between 0.01 V and 3.0 V (vs. Li^+^/Li). Consistent with the CV plots, during the first discharge step, a small voltage plateau at about 1.4 V was indicative of the formation of SEI film. The two obvious slope platforms located at 0.6 V and 0.1 V are associated with the alloying process of Cu_6_Sn_5_ to Li_2_CuSn and Li_2_CuSn to Cu_6_Sn_5_, respectively [26,32,33]. After the first cycle, the voltage plateau becomes two sloped regions, which indicates the occurrence of structure recombination. The GDC curves almost coincide with the increasing cycling number, illustrating the superior cycling stability of the Cel-CS electrode.

**Figure 5:**
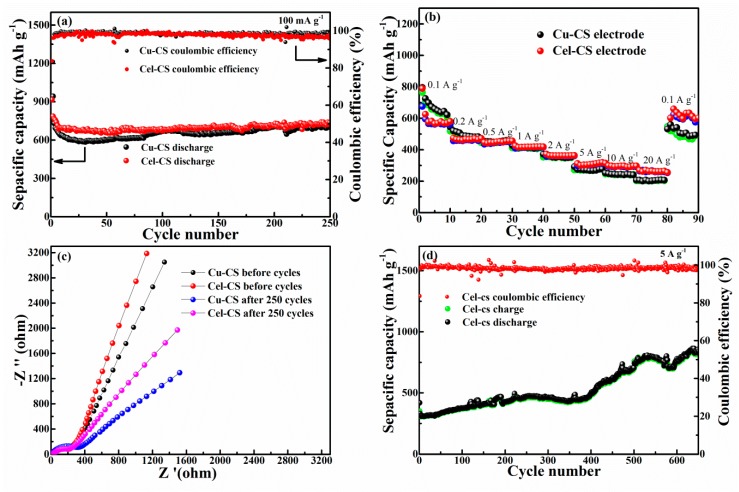
(**a**) The cycle performance of Cu-CS and Cel-CS electrodes. (**b**) The rate performance of Cu-CS and Cel-CS electrodes at different current densities. (**c**) The electrochemical impedance spectra of Cu-CS and Cel-CS electrodes after 250 cycles. (**d**) the high rate cycling performance of the Cel-CS electrode.

Figure 5a shows the GDC of the Cu-CS and Cel-CS electrodes at the current density of 100 mA·g^−1^ between 0.01 and 3.0 V (vs. Li^+^/Li). The initial discharge and charge capacities of Cel-CS electrode are 942 and 803 mA·h·g^−1^ with a coulombic efficiency of 85%. According to reports [24,28,34,35,36], the irreversible capacity is mainly attributed to the irreversible reaction, including the formation of solid electrolyte interphase (SEI) film, especially the increased interface area during the process of structure recombination and a small amount of surface oxides came from electro-deposition formed non-deintercalation Li_2_O during the first discharge process. The capacity of the Cel-CS electrode appeared stable at about 728 mA·h·g^−1^ at 250 cycles, which is close to the performance of Cu-CS electrode with the Cu current collector. Due to the Cu–Sn alloy layer of the Cel-CS electrode being directly electroplated on the separator without a current collector, the energy density of the Cel-CS electrode was much higher than that of the Cu-CS electrode. Figure 5b shows the rate performance of the Cel-CS electrode and the Cu-CS electrode at different current densities. The Cel-CS electrode clearly presents a superior rate capability. At a current density of 0.1 A·g^−1^, the Cel-CS electrodes exhibited capacities of 582 mA·h·g^−1^; even cycled at a current density of 20 A·g^−1^, a capacity of 264.7 mA·h·g^−1^ and a capacity retention of 45.5% could still be achieved, while for the Cu-CS electrode only 201.2 mA·h·g^−1^ (30.1%) was kept.

To further analyze the dynamic behavior of the Cel-CS and Cu-CS electrodes, electrochemical impedance spectroscopy (EIS) measurement (Figure 5c) was performed at 2.5 V before and after 250 cycles at a current density of 100 mA·g^−1^ [37]. All the Nyquist plots of the two electrodes consist of a semicircle in the high-medium frequency region and an inclined line in the low frequency region, which represent the charge transfer resistance (Rct) on the electrode/electrolyte interface and the Warburg impedance (W) relating to the Li^+^ diffusion, respectively. Before cycling, profiting by the smaller particle size and tighter structure, the Cel-CS electrode demonstrates the similar semicircle diameter and larger slope, implying a closed charge transfer resistances and faster Li^+^ mobility during the cycling process. After 250 cycles, the Rct and W of the Cel-CS showed an increasing trend to a smaller extent, while that of Cu-CS showed an obvious increase, implying better structure stability of the Cel-CS electrode. To illustrate the superior high rate performance of the Cel-CS electrode, the cycle curves at 5 A·g^−1^ were performed in Figure 5d. After 700 cycles, the capacity of Cel-CS electrode increased from 308 to 938 mA·h·g^−1^, which is much higher than previously reported [16,22,32,38]. The phenomenon of capacity increase can be assigned to the pseudocapacitive contributions caused by interfacial storage, which have been studied by many groups [16,39,40,41]. It can be inferred that with the increasing cycle number, the structure recombination of Sn nanoparticles occurs repeatedly, and the active particles are gradually powdered into fine grains and are accompanied by a large number of grain boundaries. Part of the lithium is stored on the increasing interface of grain during the discharge/charge process providing extra capacity.

**Figure 6:**
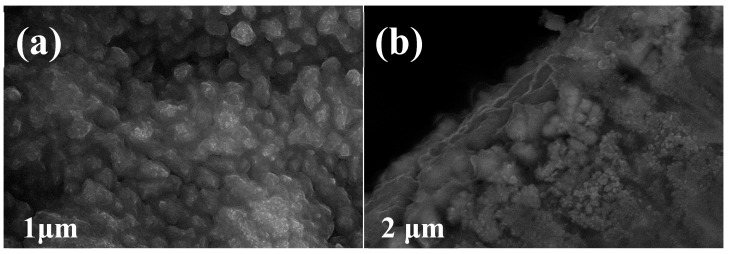
The plane (**a**) and section (**b**) FESEM images of the Cel-CS electrode after 250 cycles.

Figure 6a shows the plane FESEM images of the Cel-CS electrode after 250 cycles. Compared with the Cel-CS electrode before cycling (Figure 2a), the entire area of the Cel-CS electrode is coated with a thin layer of SEI film, which can improve the structure stability by decreasing the side reaction between the active material and electrolyte and buffering the volume expansion. In Figure 6b, consistent with our previous inference, a remarkable morphology change with particle pulverization and structural expansion was observed, implying structure reconstruction and grain refinement, which can not only buffer the volume expansion and enhance the cycle stability; but also shorten the Li^+^ diffusion path, providing faster kinetics and resulting in better rate performance.

## 4. Conclusions

In summary, a novel integrated structure electrode (Cel-CS electrode) was designed and synthesized by electrodepositing alloy anode materials on the separator. The integrated structure of the Cel-CS electrode not only greatly simplifies the battery fabrication process and increases the energy density of the whole electrode, but also buffers the volume expansion and enhances the cycle stability. The Cel-CS electrode exhibits excellent cycle performance and superior rate performance. A capacity of 728 mA·h·g^−1^ can be achieved after 250 cycles at the current density of 200 mA·g^−1^. Even cycled at a current density of 6 A·g^−1^ for 650 cycles, the Cel-CS electrode still showed a specific capacity of 938 mA·h·g^−1^, which illustrated the potential application prospects of the Cel-CS electrode, with higher energy density and power density, on MLIBs.
